# The Infant Gut Microbiome: Evidence for Obesity Risk and Dietary Intervention

**DOI:** 10.3390/nu7042237

**Published:** 2015-03-31

**Authors:** Petya T. Koleva, Sarah L. Bridgman, Anita L. Kozyrskyj

**Affiliations:** 1 Department of Pediatrics, University of Alberta, Edmonton, AB, T6G 1C9, Canada; E-Mails: koleva@ualberta.ca (P.T.K.); sarah.bridgman@ualberta.ca (S.L.B.); 2 School of Public Health, University of Alberta, Edmonton, AB, T6G 1C9, Canada; 3 Community Health Sciences, University of Manitoba, Winnipeg, MB, R3E 0W3, Canada

**Keywords:** infant, nutrition, microbiome, human milk, prebiotics, probiotics, atopy, overweight

## Abstract

Increasing globally, particularly in children, obesity is a serious public health issue and risk factor for overweight and metabolic disease in later life. Both in experimental animal and human studies, advances in gene sequencing technologies have yielded intriguing possibilities for the role of the gut microbiome in later development of overweight status. Before translating study findings into practice, we must first reconcile inconsistencies between animal experimentation, and human adult and infant studies. Recent evidence for associations with gut microbiota and infant weight gain or child weight status, implicate *Bacteroides* and *Lactobacillus* species. Dietary manipulation with human milk and pre/probiotic formulations holds promise for preventing obesity.

## 1. Introduction

Childhood obesity is recognized as one of the most serious global health issues in our society today. Affecting approximately 43 million preschool children, the prevalence of overweight and obesity has increased over the last two decades to 6.7% in 2010 [1]. Obese children are more likely to be obese in adulthood and are at greater risk of premature death and adverse health outcomes in later life [2]. The etiology of obesity is complex and involves lifestyle factors that are challenging to modify. Attention has therefore focused on preventative strategies and the identification of modifiable prenatal and early life exposures associated with overweight risk in childhood. Over the last decade, novel evidence from animal and human studies have identified associations between our intestinal bacteria (collectively known as our gut microbiota) and host metabolism and obesity [3,4,5]. Infancy is a critical period in the development of the commensal gut bacteria, with a gradual increase in colonization with the Bacteroidetes phylum from the time of birth. Initial colonization, especially with members of this phylum, is influenced by a number of early life exposures including birth mode, infant nutrition and antibiotic use [6,7,8]. The introduction and wider use of next generation sequencing techniques and metabolomic technologies has increased our ability to study the development of the gut microbiota, its metabolic functions and its association with later health outcomes during this important early life time period.

**Figure 1:**
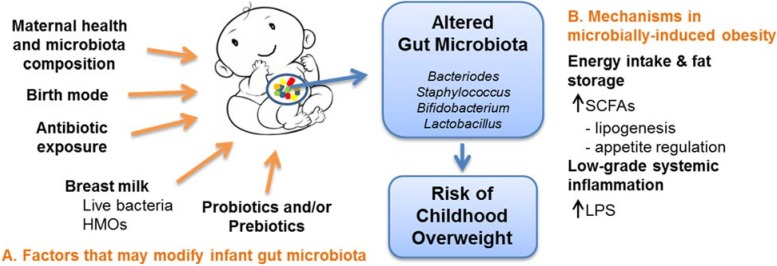
Pre and postnatal exposures demonstrated to modify the infant gut microbiota (**A**) and hypothesized to impact childhood obesity risk through microbial induced mechanisms (**B**).

The following review summarizes the current research on the association between infant gut microbiota and obesity, as well as potential early life dietary strategies to modify its composition and prevent obesity (Figure 1).

## 2. Evidence Implicating Gut Microbiota in Obesity

### 2.1. Evidence from Animal Studies

The first evidence for an obesogenic gut microbiota profile comes from animal studies by the Gordon laboratory at Washington University. Using leptin-deficient *ob/ob* mouse model and Sanger sequencing, Ley *et al.* [9] reported that *ob*/*ob* mice possessed reduced abundance of Bacteroidetes and concomitantly higher proportions of Firmicutes relative to their lean counterparts. This higher ratio of Firmicutes to Bacteroidetes in obese mice was later confirmed by Turnbaugh *et al.* [4,10] in a study with a high-fat-diet-induced obese model, using both traditional Sanger and 454 pyrosequencing technologies. The high-fat diet intervention was associated with a bloom in a single clade of Firmicutes phylum, *Mollicutes* [10], which was later re-classified as *Erysipelotrichaceae* [11]. The *Mollicutes* group was reduced by a weight loss diet; transplantation of the *Mollicutes*-enhanced microbiota to lean germ-free recipients promoted greater fat deposition than transplantation from lean donors [10]. More recently with humanized gnotobiotic mice fed a high-fat diet, Turnbaugh *et al.* demonstrated that diet-induced obesity was associated with higher proportions of the Firmicutes class, Erysipelotrichi, and lower abundance of Bacteroidetes phylum [12].

Publishing on the Zucker *fa/fa* rat model (with a defect in the leptin receptor), another research group found that genera *Bifidobacterium* and *Atopobium* were significantly less abundant in obese *fa*/*fa* animals compared to the non-obese rats, in conjunction with significantly higher levels of the *Clostridium* cluster XIVa and *Lactobacillus* group [13]. On the other hand, using FISH for microbial enumeration, Cani *et al.* reported a reduction in the *Clostridium* cluster XIVa, namely of *Clostridium coccoides* group, along with lower *Bifidobacterium* and *Bacteroides* levels in mice fed high-fat diet [14]. Further evidence implicating the gut microbiota in obesity comes from Murphy *et al.*’*s* pyrosequencing study of seven week old obese (*ob*/*ob*) and lean (*+/+*) mice, and mice fed a high-fat diet [15]. They reported a progressive increase of Firmicutes levels in both *ob*/*ob* and high-fat fed mice; fecal proportions of Bacteroides phylum decreased overtime in all groups, but statistical significance was reached only for the obese group [15].

Animal models have provided evidence of a novel link between gut microbiota and obesity. As discussed in this section, several publications of animal model studies have shown differences in the composition of gut microbiota relating to energy metabolism, and identified changes in the ratio of Firmicutes/Bacteroidetes between the lean and obese state. Experimental models have provided a powerful preclinical platform for investigating the role of gut microbiota in obesity and have set the stage for research into understanding compositional differences in intestinal bacteria between lean and obese individuals.

### 2.2. Discrepancies between Animal Models and Humans

Rodents have been widely used as animal models for different human diseases, and likewise for obesity; the reason behind their widespread use in biomedical research is the relatively close physiologic similarity with humans. However, the anatomy and morphology of the human gastrointestinal tract have some important differences in comparison with the murine system [16]. In 2014, Nguyen *et al.* published a comprehensive overview of the differences between animal models and human studies [16], and only the highlights will be discussed here.

Unlike humans, in whom bacterial fermentation of non-digestible dietary compounds takes place in the colon, rodents have a well-developed cecum where fermentation occurs. The human intestinal tract is also characterized by a relatively longer small intestine and by the presence of an appendix, which is missing in rodents. In contrast to humans, the stomach of rodents consists of glandular and non-glandular parts, whereby the non-glandular portion is lined with stratified squamous epithelium for the storage and digestion of food [17]. There are also a number of differences in the abundance and distribution of essential epithelial cells, such as goblet and Paneth cells, between humans and mice [16].

In addition to physiologic differences, the microbial composition of the intestinal system also varies between humans and rodents. For instance, lactobacilli are the dominant bacterial group in the stomach of rodents and they colonize the stratified squamous epithelium [18]. *Lactobacillus* group comprises up to 25% of the murine intestinal microbiota compared to lactobacilli in the human gut, which are mainly allochthonous members obtained from food [18]. Based on phylum levels, the murine intestinal microbial community is similar to that of humans, with Firmicutes and Bacteroidetes dominating [9,19,20]. However, Ley *et al.* has reported that 85% of the bacterial taxa in the cecal microbiota of mice represent genera that have not been detected in humans [9]. Although murine models have greatly improved our understanding of the role of gut microbiota in obesity and other metabolic disorders, extrapolation to humans requires further confirmation in clinical trials.

### 2.3. Epidemiologic Evidence from Adult Human Studies

In the comprehensive systematic review and meta-analysis by Angelakis *et al.* [21], several 16S rRNA gene sequencing studies have reported lower or higher abundance of Bacteroidetes phylum, a higher abundance of the Firmicutes phylum and higher concentrations of lactobacilli in the gut microbiota of obese and overweight adults compared to lean individuals. Some of these studies will be presented in detail here.

Consistent with animal models, Ley *et al.* reported higher proportions of Firmicutes and relatively fewer Bacteroidetes in 12 obese volunteers compared to lean controls using high-throughput sequencing technique [20]. A decrease in Bacteroidetes phylum was also confirmed in another study by Armougom *et al.*, who compared the fecal microbiota of 20 obese subjects, nine patients with anorexia nervosa and 20 normal-weight healthy controls [22]. In addition to the reduction in Bacteroidetes, higher concentrations of *Lactobacillus* species belonging to the Firmicutes phylum were found in obese patients in comparison with lean and anorexic subjects. Later studies by Raoult’s group reported that some species of *Lactobacillus* group (*L. reuteri*) were associated with obesity, while certain *Bifidobacterium* species and *Methanobrevibacter smithii* were negatively correlated with body mass index (BMI) [23,24].

The composition of gut bacterial species varies greatly between individuals [25]; however, microbial profiles are similar among family members. Thus, monozygotic or dizygotic twins discordant for obesity provide an attractive model for studying the association of intestinal bacteria with obesity [26,27]. In contrast to aforementioned studies, Turnbaugh *et al.* observed a different compositional structure of the fecal microbial community of 154 monozygotic and dizygotic twin pairs concordant for leanness or obesity, and their mothers [5]. Their study revealed a low abundance of Bacteroidetes and Actinobacteria in obese individuals compared to their lean counterparts, but no significant differences in proportions of Firmicutes were detected. Furthermore, microbial diversity was also reduced in obese subjects, a finding which was not reproduced in Ley *et al.*’s study [20].

The changes in the proportions of Firmicutes and Bacteroidetes phyla with regard to human obesity are conflicting [28,29,30]. Zhang and colleagues applied 454 pyrosequencing to examine the gut microbiota composition of three individuals with normal weight, three obese subjects and three patients with gastric-bypass surgery [30]. The study demonstrated that Firmicutes were dominant in both lean and obese individuals, and significantly decreased in the post-gastric-bypass subjects; however, abundance of *Prevotellaceae*, a family belonging to Bacteroidetes, was significantly enriched in obese volunteers. Another study with a larger sample size (30 lean, 35 overweight and 33 obese subjects) by Schwiertz *et al.* also showed reverse ratios of Firmicutes to Bacteroidetes in obese individuals compared to lean controls [29]. Levels of the genus *Bacteroides* were higher, whereas numbers of clostridial clusters IV and XIVa belonging to Firmicutes were lower in overweight and obese subjects than in lean volunteers. The changes in Firmicutes/Bacteroidetes ratio in the gut microbiota in human obesity were also questioned by Duncan and colleagues who demonstrated that abundance of the genus *Bacteroides* did not differ between obese and lean subjects, and did not change significantly after eight weeks of intervention on carbohydrate-restricted diets [28]. Statistical reduction was attained in the fecal microbiota of obese subjects on weight loss diets for the *Roseburia* and *Eubacterium* groups, which are butyrate-producing bacteria, belonging to Firmicutes phylum. Overweight women at 24 weeks of pregnancy also had decreased copy numbers of Bacteroidetes along with *Bifidobacterium* in their fecal microbiota, but had elevated numbers of *Staphylococcus* (Firmicutes phylum), and certain Proteobacteria (*Enterobacteriaceae* and particularly *Eshcerichia coli*) [31]. Correspondingly, Collado *et al.* reported an increase in *Staphylococcus* species in overweight pregnant women, but surprisingly this was in conjunction with an increase in *Bacteroides* counts [32].

Human studies conducted to date indicate that obesity may be associated with reduced bacterial diversity and shifts of intestinal bacteria at the phylum level; however, discrepancies exist in the directionality and relevance of the Firmicutes to Bacteroidetes ratio in obesity. Conflicting findings could be explained by the heterogeneity in age, genetic background, ethnicity, lifestyle and diet of subjects across studies, factors that are well controlled in animal models. Reported shifts at the phylum level do not completely capture the compositional changes of the gut microbiota associated with obesity. Future studies in human obesity should be conducted at lower taxonomic levels (family, genus, and species) and ensure adequate adjustment for confounding variables.

### 2.4. Epidemiologic Evidence from Infant Studies

Similar to studies in adults, gut microbiota compositional differences are also evident between overweight and lean children. In Karlsson’s case-control study of four to five year olds, members of the *Enterobacteriaceae* family were more often identified by qPCR in fecal samples of overweight *versus* normal weight children [33]. Using culture and molecular methods, Bervoets *et al.*’s cross-sectional study of older children (age 6–10) reported *Bacteroides fragilis* to be more prevalent in gut microbiota of children with a higher body-mass index (BMI) [34]. However, other *Bacteroides* species such as *B. vulgatus* were less abundant and lactobacilli were more prevalent in overweight children. Of note in the Bervoets study is that fecal lactobacilli concentrations in children were correlated with a serum marker of inflammation, CRP (C-reactive protein).

It is likely that obesity-related gut dysbiosis has its origins during infancy, as early as 3–6 months after birth, at a time when first colonizers of gut microbiota lay the foundation for subsequent colonization by anaerobes from the Bacteroidetes phylum [35,36]. To date, five epidemiological studies have published evidence on associations between infant gut microbiota and infant weight gain or later child overweight (Table 1). Two are nested case-control studies of children, matched on birth mode, gestational age, birth weight, probiotic intervention group, breastfeeding duration, antibiotic use and atopic disease status, selected from a prospective follow-up of high risk (for allergy) infants [37,38], who had been randomized to pre- and postnatal probiotic supplementation [39]. Using FISH flow cytometry and qPCR methods, Kalliomaki *et al.* reported lower bifidobacterial numbers and higher counts of *Staphylococcus aureas* in fecal samples at six and 12 months in children classified as overweight (*n* = 22) at age 7 than those of normal weight (*n* = 17) [37]. Overweight children tended to have lower counts of lactobacilli but higher counts of *B. fragilis* at six months of age. In a smaller sample of children from the same study, Luoto *et al.* found that bifidobacterial numbers also tended to be lower in fecal samples obtained at three months of age in children who became overweight at age 10 compared to those who did not, although this difference did not reach significance [38].

In a general population cohort study of 138 vaginally-delivered full-term infants, higher *B. fragilis* in gut microbiota at age 3–26 weeks and lower staphylococcal concentrations (as measured by culture-based techniques) were correlated with a higher BMI z-score in preschool children between one and three years of age [40]. Regression analyses were used to control for known risk factors of childhood overweight, including maternal BMI and smoking status, birth weight, breastfeeding status and infant use of antibiotics. On the other hand, prospective follow-up of 218 full-term infants, delivered vaginally and not exposed to antibiotics, found early detection of *Bacteriodes* species (according to DNA cloning methods) in fecal samples at one month of age to be associated with a reduced growth trajectory over the first six months of life in male infants only; the presence of *Staphylococcus* species at Day 4 was associated with expected growth in both males and females [41]. These findings were independent of maternal BMI and other pregnancy complications, fetal growth and birth weight, and breastfeeding status.

More recently, findings on intestinal microbiota composition and infant weight gain has been reported from the large prospective KOALA Dutch birth cohort study [42] that recruited pregnant women with conventional and anthroposophic lifestyles. Gut colonization with *B. fragilis* at one month postpartum in the conventional cohort was significantly associated with a higher BMI z-score, measured repeatedly between one and 10 years, but only among infants with a low fibre intake at age 4 after adjustment for a myriad of confounding factors, including pre-pregnancy overweight, birth mode, breastfeeding duration and caloric intake at age 4. Among colonized children, *B. fragilis* counts were positively associated with BMI z-score in children in the conventional high-fibre diet subcohort and negatively associated with BMI in the low fibre and alternative subcohorts. Linear regression analysis also showed that children in the conventional sub-cohort, colonized with *C. difficile* at one month, had a lower BMI z-score at eight and a half years of age.

In addition, epidemiological studies of antibiotic use in infancy have also provided indirect evidence for the role of the gut microbiota in the development of overweight, independent of established obesity risk factors [43,44,45,46,47]. Data from two large birth cohorts, the Danish National Birth Cohort and the UK Avon Longitudinal Study of Parents and Children, found modest increases in risk for overweight at age 7 years and 38 months, respectively, with antibiotic exposure before six months of life [43,47]. A large US study of electronic health records from 65,000 children reported an association between frequent courses of antibiotic treatment within the first two years after birth and risk for obesity (95th percentile BMI) between age 1 and 5, with a stronger effect for broad spectrum antibiotics [45]. In a smaller prescription database-linkage Canadian study, Azad *et al.* reported significantly greater odds of childhood overweight at age 9 and 12 years with exposure to antibiotics during the first year of life, but in boys only [44]. This study also found an association between infant antibiotic exposure and later central adiposity (measured waist circumference), thought to be a better predictor of cardiovascular outcomes than BMI-based measures of overweight. A similar sex-specific effect of infant antibiotic exposure and BMI was reported in a large cross-sectional study by Murphy *et al.* with data from the International Study of Asthma and Allergies in Childhood [46].

**Table 1:** Associations between gut microbial community and infant overweight status.

Authors and Year of Publication	Study Design	Participants (Exclusion Criteria)	Microbiota Profiling Time Point and Method	Overweight Assessment	Main Significant Findings Associated with Overweight	Confounding Variables Considered in Design/Analysis
Scheepers *et al.* 2014 [42]	Prospective general and anthroposophic cohort	909 infants (Preterm birth before 37 weeks gestation, twins, presence of congenital abnormalities relating to growth, use of antibiotics before fecal collection)	1 month Quantitative qPCR: Bifidobacteria, *Bacteriodes fragilis*, *Clostridium difficile*, *Escherichia coli*, Lactobaccili, total bacterial counts	Age and gender standardized BMI z-scores from parent report weight and height at 7 time points between ages 1–10 years	+ve *B.fragilis* colonization (*only* in children with low fibre intake at age 4 years in conventional subcohort)	Analysis controlled for gender, place and mode of delivery, birth weight, age at collection of fecal sample, maternal smoking during pregnancy, type of infant feeding in the first month, duration of breastfeeding, maternal education, and total bacterial counts.
					↑ *B. fragilis* counts in conventional high-fibre diet subcohort	
					↓ *B. fragilis* counts in low fibre subcohort and alternative subcohort	
White *et al.* 2013 [41]	Prospective general cohort	218 infants (Preterm (GA<253 days), term infants born via cesarean section and term infants born vaginally but exposed to antibiotics before Day 4 were excluded from analysis)	4, 10, 30, 120 days BLAST: *Enterococcus* spp., *Lactobacillus* spp., *Lactobacillus paracasei/casei*, *Staphylococcus* spp., *Streptococcus* spp., *Clostridium* spp., *Lachnospiraceae* spp., *Veillonella* spp., *Pseudomonas* spp., *Escherichia coli*, Enterobacteriaceae other than *E.coli*, Gammaproteobacteria, *Varibaculum* spp., *Bifidobacterium longum*, *Bifidobacterium bifidum*, *Bifidobacterium breve*, *Bifidobacterium* spp., *Bacteriodes fragilis*, *Bacteroides* spp.	Difference in weight-for-age z-score from birth to 6 months from parent report weight	−ve *Bacteriodes spp*. colonization at Day 30 (males only)	Analysis controlled for antibiotic use after Day 4, sex, use of milk substitutes, maternal smoking, and parity.
Luoto *et al.* 2011 [38]	Nested matched case- control	15 overweight or obese and 15 normal weight children with family history of atopic disease	3 months FISH: *Bacteriodes-protovella* group, *Bifidobacterium* genus, *Clostridium histolyticum* group, *Lactobacillus-Lactococcus-Enterococcus* group and total counts	BMI at 10 years from parent reported weight and height	↓ bifidobacteria numbers (NS)	Matched for sex, gestational age, BMI at birth, mode of delivery, probiotic intervention, and duration of breastfeeding.
Vael *et al.* 2011 [40]	Prospective general cohort	138 infants (Preterm birth, delivery by cesarean section)	3, 26 and 52 weeks Cultures: *Bacteriodes fagilis, Bifidobacterium, Lactobacillus, Enterococci, Enterobacteriaceae, Clostridium, Staphylococcus*	BMI at 12, 18, 24, 30, 36 months from parent reported weight and height	↑ *B. fragilis* concentration at 3 and 26 weeks	Analysis controlled for maternal BMI, formula or breastfeeding, antibiotic use in infancy, SES, maternal smoking status, birth weight.
					↓ *Staphylococcus* concentration at 3 and 52 weeks	
					↓ *Staphylococcus/B.fragilis* ratio at 3 weeks	
Kalliomaki *et al.* 2008 [37]	Nested matched case-control	25 overweight or obese and 24 normal weight children with family history of atopic disease	6 and 12 months FISH/FISH-FCM: *Bacteriodes- protovella* group, *Bifidobacterium* genus, *Clostridium histolyticum* group, *Lactobacillus-Lactococcus-Enterococcus* group and total counts. qPCR: *Bifidobacterium* genus, *Bifidobacterium adolescentis, Bifidobacterium bifidum, Bifidobacterium breve, Bifidobacterium longum, Bacteriodes fragilis, Staphylococcus aureas.*	BMI at 7 years from parent reported weight and height	↓ bifidobacterial numbers	Infants matched for gestational age, BMI at birth, mode of delivery, probiotic intervention, duration of breastfeeding, antibiotics in infancy, and frequency of atopic diseases and sensitization at 7 years of age.

Abbreviations: BLAST, Basic Local Alignment Search Tool; BMI, Body Mass Index; FISH-FCM, Fluorescent *in situ* hybridization coupled with flow cytometry; qPCR, quantitative polymerase chain reaction; NS, non-significant; SES, Socio-economic status.

To sum, the current evidence from prospective studies investigating the association between infant gut microbiota and later childhood overweight is limited. Studies by Vael *et al.*, Bervoets *et al.* and Scheepers *et al.*, as well as other human studies in adults, point to a role for *Bacteriodes* spp., in particular *B. fragilis*, in weight development in early life [20,22,34,40,42]. Gut lactobacilli and staphylococci may also be important for regulating growth in infants and young children. It is likely that growth is sensitive to perturbations in diet or other environmental factors during critical windows of microbiota development. For example, overweight during pregnancy is reported to lower counts of infant gut *Bacteroides* spp. at one month but not six months of age. Also, Vael *et al.* and White *et al.* reported stronger correlations between infant growth and *B. fragilis* counts at <26 weeks but not later during infancy.

The use of targeted and older microbiota profiling (culture and molecular) techniques also proves challenging for comparative evaluations. Moreover, with an incomplete view of the intestinal microbiota, it is difficult to assess if certain bacteria phyla or species, such as *B. fragilis* are indeed key obesogenic microbes or simply an indicator of other aberrations in bacterial taxa that have a greater influence on early weight gain. Although current studies have adjusted for important confounding factors such as birth method, breastfeeding status and antibiotic use, the potential for bias remains by way of differences in design (prospective *versus.* nested case-control), selection of study subjects (general population *versus* high risk for atopy), and variable time points for fecal sampling and overweight assessment. Large, longitudinal cohort studies which employ next-generation sequencing to profile the whole gut microbiota community in fecal samples collected at multiple time points and which collect detailed information on early and later childhood covariates (such as diet and level of activity) are required for greater understanding of the development of the infant microbiota and its association with childhood overweight. Since it is quite likely that infant gut microbiota dysbiosis is in the pathway between a perinatal exposure of interest and overweight development, it is also imperative that future analyses employ SEM or path techniques.

## 3. Mechanisms in Microbially-Induced Obesity

Most of the proposed mechanisms connecting gut microbiota to obesity risk and metabolic disorders are based on findings from animal models. A first hypothesis that gut microbes have a role in dietary energy harvest and fat storage came from the observation that germ-free mice (raised without the presence of microorganisms) had less total body fat compared to their conventionally raised counterparts, even though mice with a normal gut microbiota consumed 29% less food [3]. Further studies showed that conventionalization (the process of introducing microbiota through intestinal contents) of germ-free mice also led to a 60% increase in their total body fat and a decrease in insulin sensitivity, despite lower food intake [3,48]. The same studies demonstrated that gut colonization increased the ability of the host to extract energy from indigestible complex plant polysaccharides in the diet [3] and to regulate energy storage as triglycerides [48].

The more recent study of fecal microbiota transplantation in adult female twin pairs discordant for obesity into germ-free mice has further clarified the casual role of gut bacteria in obesity [49]. Transplanting microbiota from the obese twin caused gains to total body and fat mass; obesity-associated metabolic phenotypes were also transmitted. The obese gut microbiota phenotype is rich in genes involved in energy harvest and metabolism. As evident in the studies by Turnbaugh *et al.*, the gut microbial community of obese mice is enriched with genes coding for enzymes that utilize non-digestible dietary carbohydrates to produce the short-chain fatty acids (SCFAs): acetate, propionate and butyrate [4,10]. In humans, 95% of synthesized SCFAs are absorbed in the colon and contribute to *de novo* production of lipids by serving as energy substrates or signaling molecules [50,51,52].

The energy extraction hypothesis was recently supported by evidence from humans in Jumpertz *et al.*’s study of 12 lean and nine obese adult males fed various caloric diets [53]. In response to alterations in caloric intake, an increase in energy harvest of ~150 kcal was associated with an increase in gut microbial abundance of the phylum Firmicutes and a reduction in Bacteroidetes phylum. Their study also demonstrated that the gut microbial community can adapt to an enriched diet, modulate nutrient assimilation and contribute additional calories to the host. Payne *et al.*’s study of fecal metabolites in overweight *versus* normal children also supports the increased energy harvest hypothesis [54]. Overweight children had much lower levels of intermediate metabolites such as lactate, yet higher levels of butyrate, a by-product of lactate-utilizing microbiota. Their metabolite profile suggested exhaustive substrate utilization by obese gut microbiota.

Animal and human studies have uncovered other mechanisms linking gut microbiota to obesity. Intestinal bacteria and their SCFA metabolites can influence gut satiety hormone levels (such as glucagon-like peptide 1 and peptide YY) that convey a sense of “fullness” and regulate appetite [55,56]. These satiety hormones are also able to alter host signaling pathways involved in lipogenesis in the liver [57]. More recently, it has also been suggested that gut microbiota can contribute to the low-grade systemic inflammatory state of obesity by regulating innate immunity and inflammatory signaling. This hypothesis involves the translocation of bacterial lipopolysaccharides (LPS, a component of the cell wall of Gram-negative bacteria) from the intestinal lumen to the circulation, which initiates inflammation through activation of Toll-like receptors on macrophages and intestinal epithelial cells [14,58]. Cani and co-workers studied the role of LPS as a trigger for the development of obesity in a murine model on high-fat diet [14]. Feeding a high-fat diet to mice significantly increased plasma LPS levels, weight gain and insulin resistance in these mice [14,58]. An elevation in circulating LPS levels and subsequent obesity was also observed in mice fed a control diet if they were directly infused with LPS for four weeks.

In sum, it is plausible that the human gut microbiota could make a significant contribution to obesity development involving the following pathways: increasing energy recovery from the diet, modulating liver lipogenesis, regulating appetite through gut satiety hormones, or activating innate immunity through LPS-Toll-like receptors. Since these pathways all have been linked to the gut microbial community, dietary manipulation of this “organ system” has the potential to reduce risk for obesity.

## 4. Dietary Manipulation of the Infant Gut Microbiota in Preventing Obesity

The establishment of our intestinal microbiota begins at birth and in some infants, colonization occurs *in utero*. Throughout the first year of life, microbial numbers and diversity increase, converging towards the microbiota of the adult. Mode of delivery, infant diet, and maternal or infant antibiotic treatment are the main early life exposures that influence microbial colonization and development in infancy [6,7,8]. Once established, the gut microbial community is relatively stable throughout adult life; however, bacterial infections, antibiotic interventions, lifestyle and changes in diet may contribute to shifts in bacterial composition. The development of the gut microbiome during infancy plays a crucial role in the maturation of immunologic and metabolic pathways [59]. Compelling evidence supports the concept that shifts in this complex microbial system that occur early in life may confer an increased risk for developing obesity later in life [34,40,42].

Manipulating the gut microbiota at this early stage might offer new therapeutic possibilities for prevention and/or treatment of obesity. A review on fecal transplantation, as a method to manipulate gut microbiota, has been published by West *et al.* [60]; to date fecal transplantation has been tested in animal models and human trials of adults with *C. difficile* diarrhea. No trials have been conducted in infants or preschool children. Hence, in this review, we have limited our discussion on gut microbiota manipulation in infants to intervention by human milk and pre/probiotic formulations. These interventions and associated challenges are detailed below.

### 4.1. Human Milk

Human milk is uniquely adapted to infants, providing complete nutrition during the first six months of life. Breastfeeding has been associated with a number of health benefits including a reduced risk of later overweight [61,62,63]. The protective effect of breastfeeding may in part be mediated through its effect on infant microbiota colonization and development.

As well as providing essential nutrition for infant growth and development, human milk contains a large proportion of bioactive compounds important in the stimulation of the immune system and intestinal microbiota [64,65]. Human milk oligosaccharides (HMO) represent the third largest component of human milk, present in similar quantities to protein [66,67]. HMOs are complex sugars that resist digestion by the stomach and reach the small intestine and colon intact where they are metabolized by selective intestinal microbiota, increasing their numbers and function within the gut [68,69]. The metabolism of HMOs by certain bacteria leads to the production of SCFA which reduce the pH of the intestinal lumen altering the microbiota profile and inhibiting pathogen growth [70,71]. As discussed above, studies have revealed that SCFA may contribute to dietary energy harvest, modulate host adiposity, and alter gene expression of host satiety hormones [51,52,55,57].

In addition to providing substrates for microbiota metabolism, there is overwhelming evidence from several recent studies demonstrating the presence of live bacteria in human milk [72,73,74]. Such studies have recently been summarized by McGuire & McGuire and demonstrate the large diversity and richness of the human milk microbiome which includes, but is not limited to, *Bifidobacterium*, *Lactobacillus*, *Staphylococcus* and *Streptococcus* [75]. Traditionally, the presence of bacteria in human milk was thought to be a result of contamination from maternal skin. However, this has been disputed by studies that have shown that orally administered bacteria given to lactating women can be subsequently detected in human milk [76,77] and knowledge that certain genera, such as bifidobacteria, are strict anaerobes and therefore their presence in human milk is unlikely due to contamination by maternal skin. This suggests that contamination is not the only source of bacteria in human milk and has led to speculation that bacteria are able to migrate from the intestine to the mammary glands [78].

Accordingly, differences in early gut microbiota between breast and formula fed infants have been observed in several studies. Using culture-independent techniques, Penders *et al.* found that exclusively formula-fed infants were more often colonized with *E. coli*, *C. difficile*, *Bacteroides*, and lactobacilli, compared with breastfed infants [8]. More recently, Azad *et al.* [6] found the gut microbiota of non-breastfed infants had increased richness of species, with overrepresentation of *Peptostreptococcaceae*, *Verrumicrobiaceae*, and *C. difficile*.

### 4.2. Challenges with Breastfeeding Interventions

As discussed previously, breastfeeding confers a number of health benefits to infants including a reduced risk of obesity, which may in part, be modulated through the effect of human milk on the developing infant gut microbiome. There is no doubt that breastfeeding is the preferred food source for the majority of infants. However, it is important to remember that human milk is not uniform and can differ significantly between mothers [79], including the presence of specific bacterial taxa [75]. Some of this variation may be due to methodological issues; however, recent studies have shown that certain maternal factors, including weight status, atopy and birth mode can influence the composition of the human milk microbiota that may in turn effect the establishment of the infant microbiome and later health [80,81,82].

Cabrera-Rubio *et al.* characterized the microbiota present in 18 mothers with varying BMI, pregnancy weight gain and delivery mode [80]. Lower microbial diversity was observed in colostrum (first breast milk) and one month breast milk samples from obese mothers compared to normal weight mothers. Data from qPCR analysis revealed higher bacterial counts, higher *Staphylococcus* and *Lactobacillus* and lower *Bifidobacterium* amounts in breast milk of obese *versus* normal weight mothers over the first six months of breastfeeding. Excessive pregnancy weight gain was associated with similar patterns including higher counts of the genus *Staphylococcus* as well as *Staphylococcus aureas* in one month breast milk samples and higher counts of *Lactobacillus* and lower *Bifidobacterium* spp. in six month samples. Differences in breast milk microbiota were also observed according to birth mode, most distinctly in those mothers giving birth by elective caesarean section.

Allergic status of mothers has also been shown to alter human milk microbiota [81]. In a study of 61 mother—infant pairs, human milk samples taken at one month from allergic mothers had significantly lower amounts of bifidobacteria compared to samples from non-allergic mothers. In parallel, lower bifidobacteria counts were observed in fecal samples of infants from allergic mothers compared to those from non-allergic mothers. Epidemiological evidence exists that infants from obese mothers are at greater risk of asthma and wheeze [83] and there is some evidence that the opposite relationship may also occur [44]. Differences in breast milk microbiota (influenced by maternal health status) and its subsequent impact on the developing infant microbiome may be one mechanism by which this association occurs.

Whilst breastfeeding should be encouraged, these data provide evidence that the simple promotion of breastfeeding to establish a “healthy” gut microbiota in infancy is complicated by the variability of bacterial composition of human milk due to maternal health factors. Indeed, breastfeeding by obese mothers may in fact propagate this condition in infants through the transfer of aberrant microbiota in human milk during the early establishment of the infant microbiome. Future studies are required to investigate the effects of maternal and environmental exposures, including maternal overweight, on the human milk microbiome and these variations should be considered when investigating associations between breastfeeding, infant microbiota and later health.

### 4.3. Probiotic Interventions

Early manipulation of gut microbiota with probiotics is another therapeutic strategy to prevent or modulate obesity. Initially proposed by Fuller in 1991, probiotics are defined by the Food and Agricultural Organization of the United Nations, and WHO as “live microorganisms which when administered in adequate amounts, confer a health benefit to the host” [84,85]. The most commonly-used probiotic strains belong to the *Lactobacillus* and *Bifidobacterium* species, and several have been isolated from human milk. Their beneficial role in gut health by for example, suppressing the proliferation of pathogenic microbes, has been extensively studied [86]. More recently, adult clinical trials suggest that probiotics hold promise in the treatment of obesity [87]. Probiotics are also being tested in the treatment of obesity in children. In a randomized controlled trial of 70 overweight and obese children at age 10, Safavi *et al.* assessed anthropometric outcomes following the administration of a symbiotic, a combination product of seven probiotic strains (*L. rhamnosus*, *L. acidophilus*, *L. casei*, *L. bulgaricus*, *S. thermophilus*, *B. breve*, *B.longum*) and prebiotic fructo-oligosaccharides (FOS) [84]. A significant reduction in BMI z-scores, waist circumferences and waist-to-hip ratios was observed with synbiotic treatment compared to the placebo. Oral intervention with a probiotic mixture, containing three *Bifidobacterium* strains, four *Lactobacillus* strains and one *Streptococcus* strain, was also associated with reduced BMI in overweight children.

Probiotics have also been added to infant formula to simulate the “live bacteria” of breast milk and facilitate colonization of the infant gut [72,73,74]. Infant weight gain has been evaluated as an outcome in probiotic trials to assess product safety. In their systematic review of six probiotic formula-supplementation trials [88], Braegger *et al.* found no significant differences in infant growth between the probiotic and control arms. Probiotics studied were *B. lactis*, *S. thermophiles*, *L. helveticus*, *L. johnsonii*, *L. reuteri*, and *L. salivarius*. One trial of cow’s milk formula supplementation with *B. lactis* and *S. thermophiles* in malnourished Thai orphans, age 6–36 months, reported significant improvement in growth with the probiotic-enhanced formulas [89].

Few studies have been performed to test the effectiveness of probiotics during infancy in overweight prevention. Karlsson *et al.* evaluated cereal supplementation with the probiotic strain *L. paracasei* ssp. of four-month old infants for six months during weaning from breast-feeding [90]. Randomization to probiotic supplementation did not change the BMI-z score of infants between four and 13 months of age, or at follow-up seven to eight years later. Free-fat mass, insulin resistance, or other body composition and metabolic markers of children at age eight to nine years also did not differ between the probiotic and control groups. On the other hand, Luoto *et al.*’s randomized double-blind controlled trial documented short-term effects of a perinatal and postnatal probiotic intervention on infant growth [91]. A total of 159 pregnant women received treatment with the probiotic *L. rhamnosus* GG for four weeks before delivery, which continued for six months postnatally. Administration of *L. rhamnosus* was associated with reduced excessive weight gain in offspring during their first two years of life. Since in both of these trials, the probiotic was administered for six months postnatally, the Luoto *et al.*’s findings hint at the importance of starting probiotic treatment during pregnancy.

### 4.4. Prebiotic Interventions

The manipulation of the gut microbial community to prevent or treat obesity can also be achieved with the administration of prebiotics, such as FOS, inulin, galacto-oligosaccharides (GOS) and lactulose. Prebiotics are non-digestible food ingredients which are fermented by gut bacteria to SCFAs, and in this way, they stimulate the growth and/or activity of gut bacteria [92]. Compared to probiotics, experimental reports and human studies with prebiotics have shown more promising results in obesity management, with reductions in body weight and fat mass [93,94,95,96]. As mentioned above, Safavi *et al.* [97] found that treatment of overweight children with a synbiotic mixture of the prebiotic, FOS, in combination with seven probiotic strains was associated with a decreased BMI z-score compared to placebo. It was not clear whether these anthropometric outcomes were attributed to FOS or the combination of probiotic strains.

The prebiotic mixture of 90% GOS plus 10% FOS has been assessed to be safe when added to infant formula [98]. In the systematic reviews by Braegger *et al.* [88] and also by Rao *et al.* [99], none of the 10 identified trials individually reported that prebiotic-supplemented formulas influenced infant growth when compared to un-supplemented formulas. However, after compiling data from four of these trials into a meta-analysis (*n* = 436), weight gain (weighted mean difference 1.07 g/day, 95% CI 0.14–1.99) was significantly higher among infants fed formula supplemented with prebiotics compared to the placebo group. A concern with this meta-analysis was that data were pooled from various prebiotic-supplemented infant formulas, including extensively hydrolysed whey formulas.

Although this trial did not measure weight or growth, Holscher *et al.* studied the effect of prebiotic formulas on gut microbiota composition and production of SCFA in healthy, full-term infants [100]. Infants were randomized to receive either control formula or formula supplemented with the prebiotic mixture of GOS plus FOS (9:1 ratio); both groups were compared to exclusively breastfed infants. Fecal microbiota composition and SCFA production were similar between prebiotic formula-fed and breastfed infants, but differed from infants fed control formula. As discussed above, colonic SCFAs may serve as energy substrates for lipogenesis [57] or as signaling molecules to regulate sense of “fullness” by stimulating secretion of satiety hormones [55,56]. Further research is required to establish whether prebiotic treatment during infancy represents a realistic therapeutic strategy for preventing obesity risk.

### 4.5. Challenges with Pre/Probiotic Interventions

Whilst studies from adults and children have suggested some beneficial effects of probiotics on controlling obesity [87,97], the evidence to support the role of probiotics in preventing obesity is insufficient in the infant age group and further studies are required. Moreover, there are still many questions that need to be answered, including which probiotic strains and dosage, timing of probiotic administration, and concurrent infant diet are optimal [101].

As aforementioned, *Lactobacillus* strains are some of the most commonly used probiotics. Million *et al.* have contributed meta-analytic evidence of statistically significant weight gain with *Lactobacillus* probiotic treatment in animal models and human trials [102,103]. These findings are consistent with the use of probiotic lactobacilli in livestock agriculture as growth promoters [104]. However, weight gain was dependent on the strain of the probiotic [103]. The *L. acidophilus* strain was associated with the lowest weight gain, followed by *L. fermentum*; the largest weight gain was seen with the *L. ingluviei* strain. The *Lactobacillus* species, *L. plantarum* and *L. gasseri*, were associated with weight loss.

The degree of weight gain has also been found to vary according to the real-world context of the administered probiotic, which, as illustrated in the next two examples, involves the (infant) host. Suspecting that improved survival of breastfed infants was due to the presence of lactic acid bacteria in the infant gut, Robinson *et al.* conducted a trial in the 1950s to test the addition of *L. acidophilus* to cow’s milk formula on infant growth outcomes [105]. Indeed, formula-fed infants supplemented with *L. acidophilus* showed significantly larger weight gain than did unsupplemented formula-fed controls. However, the addition of *L. acidophilus* to infant formula had no effect on weight gain if infants were partially breastfed.

Similarly, Abrahamsson *et al.*’s randomized controlled trial of *L. reuteri* reported some interesting differences in gut microbiota composition according to breastfeeding status [106]. In addition to *L. reuteri* being detected in maternal stool and colostrum, other *Lactobacillus* species were also elevated in colostrum. These findings were viewed as evidence of maternal stool and breast milk routes of transmission of the probiotic (and other breast milk microbiota induced by the probiotic) to the nursing infant. Unexpectedly, the prevalence of *L. reuteri* declined in breast milk and newborn gut microbiota after the first week of continuous supplementation. Further, despite being detected in breast milk, gut microbiota levels of *L. reuteri* were lower among infants breastfed than those formula-fed. The reduction of *L. reuteri* in infant gut microbiota over time was interpreted as the outcome of competition from other microbiota and/or infant immune recognition of *L. reuteri*. Authors speculated that immune recognition and reduction of *L. reuteri* may have been heightened in breastfed infants receiving additional immunoglobulin A from mother’s milk.

The potential for prebiotics to be used in the prevention or management of childhood obesity is equally challenging. Human milk contains over 100 different HMOs that have been shown to vary by blood group and maternal secretor status [68]. Supplementation of non-breastfed infants with all of the identified HMOs is unachievable. Research to date has focused on a select number of prebiotics that have been shown to mimic the structure and functionality of HMOs [107]; however, it remains to be shown if these few will confer benefits relating to healthy weight gain in childhood.

## 5. Conclusions and Future Perspectives

Advances in gene sequencing technologies have yielded intriguing possibilities for the role of the gut microbiome in the development of overweight status in later life. As exciting as this literature is, and as tempting it is to quickly proceed to translating study findings into practice, we must first reconcile inconsistencies between animal experimentation and human observation. In this review, we have discussed sources of discrepant findings between animal models and human studies, as well as within human studies. Animal models make valuable contributions to our knowledge and understanding of pathways for disease. Those which most closely resemble the human, for example piglet models, and take into account covariates important to variation in the human microbiome have the greatest potential for successful translation.

We have also alerted the reader to the notion that gut microbiota compositional and metabolite biomarkers for obesity in adulthood may not be applicable to infancy, a period of substantial plasticity when gut microbiota are being shaped by the infant diet, birth events and prenatal exposures [39,40]. Evidence from studies in adults and even children may, in fact, impede our understanding of the mediating role of early gut microbiota in overweight development. Hence, it is imperative that findings from animal models, as well as those from adults, be tested in human infants. In turn, prospective follow-up studies of pregnancy cohorts which employ standardized infant fecal sample profiling techniques, and adequate sampling and adjustment for well-known covariates, are able to provide unbiased signals of microbiome-health associations for further testing in animals.

The effectiveness of dietary interventions discussed in this review—breastfeeding, and treatment with pre- and probiotic products—is dependent on the stage of gut microbiota development, infant diet, and health status of both mother and infant. As we pointed out, women with pre-existing conditions during pregnancy, such as atopy and overweight, have altered breast milk composition, which in turn, changes the gut microbial community structure of their infants. Breast milk, itself, may interact with the host system of the infant to modify the effectiveness of administered pre- and probiotics. Timing of the intervention is an important consideration. We have suggested that early microbiota development is key but how early is early? With the detection of microbes in the placenta and amniotic fluid [108], and in meconium (infant’s first stool) [109,110], development has been extended to the time of pregnancy and now subject to maternal influence. For example, Gosalbes *et al.* reported two distinct profiles of meconium microbiota composition in newborns [109]; lactic acid bacteria were more dominant in the meconium following maternal consumption of an organic food diet during pregnancy.

Finally, keystone species and their ratios may be overly simplistic biomarkers in microbiome models for overweight and metabolic disorders, and require continual testing and refinement. Even current theories on metabolic pathways may be overly simplistic, necessitating that our search for gut microbiota biomarkers for overweight to extend current paradigms of thinking. As evidenced by recent reports of sex differences in infant risk for overweight following antibiotic use [44], new theories may need to consider the influences of infant gender, ethnicity and geographic location on breastfeeding and pre/probiotic interventions aimed at preventing childhood obesity.
